# Transcriptomic response of durum wheat to nitrogen starvation

**DOI:** 10.1038/s41598-017-01377-0

**Published:** 2017-04-26

**Authors:** Pasquale L. Curci, Riccardo Aiese Cigliano, Diana L. Zuluaga, Michela Janni, Walter Sanseverino, Gabriella Sonnante

**Affiliations:** 1 Institute of Biosciences and Bioresources, National Research Council (CNR), Via Amendola 165/A, 70126 Bari, Italy; 2 Sequentia Biotech SL, Calle Comte D’Urgell 240, 08036 Barcelona, Spain; 3 Institute for Electronics and Magnetism, National Research Council (CNR), Parco Area delle Scienze 37/A, 43124 Parma, Italy

## Abstract

Nitrogen (N) is a key macronutrient representing a limiting factor for plant growth and development and affects productivity in wheat. In this study, durum wheat response to N chronic starvation during grain filling was investigated through a transcriptomic approach in roots, leaves/stems, flag leaf and spikes of cv. Svevo. Nitrogen stress negatively influenced plant height, tillering, flag leaf area, spike and seed traits, and total N content. RNA-seq data revealed 4,626 differentially expressed genes (DEGs). Most transcriptomic changes were observed in roots, with 3,270 DEGs, while 963 were found in leaves/stems, 470 in flag leaf, and 355 in spike tissues. A total of 799 gene ontology (GO) terms were identified, 180 and 619 among the upregulated and downregulated genes, respectively. Among the most addressed GO categories, N compound metabolism, carbon metabolism, and photosynthesis were mostly represented. Interesting DEGs, such as N transporters, genes involved in N assimilation, along with transcription factors, protein kinases and other genes related to stress were highlighted. These results provide valuable information about the transcriptomic response to chronic N stress in durum wheat, which could be useful for future improvement of N use efficiency.

## Introduction

As a crucial component of amino acids, proteins, nucleic acids, chlorophyll and several plant hormones, nitrogen (N) represents a key macronutrient for crop productivity^1^. Its availability influences major plant processes such as growth, development, architecture, flowering, senescence, photosynthesis, and allocation of photosynthates in plants^2^. It is well known that most diffused cereal crop plants, like wheat, rice and maize, use only 30–40% of the applied N fertilizers, while the rest remains unused causing severe environmental pollution^3^, with eutrophication of water and enrichment of NO_x_ gases in the atmosphere^4^. The newly formed and released nitrous oxide has 300 times more global warming effect than carbon dioxide^5^.

There is thus a need to sustain high productivity while decreasing the rate of N application. To this aim, it is important to obtain a better understanding of the molecular regulatory mechanisms underlying morphological and physiological acclimation to N availability in crops^6, 7^. Although both organic and inorganic N can be used by plants^8, 9^, inorganic N resources such as nitrate (NO_3_^−^) and ammonium (NH_4_^+^) are the major N forms in soil, the former being more abundant in aerobic soils, the latter the major N compound in flooded wetland or acidic soils^7^. The primary N metabolism involves several crucial steps. Nitrate is taken up and transported by low and high affinity nitrate transporter genes (*NRT1* and *NRT2*), reduced to nitrite by nitrate reductase (*NR*), and to ammonium by nitrite reductase (*NiR*). The ammonium derived from nitrate or directly taken up by ammonium transporters (*AMT*s) is then incorporated into amino acids mainly through glutamine synthetase (*GS*) and glutamate synthase (*GOGAT*) in plastids^6^. The amino group of glutamate (Glu) can be transferred to amino acids by several aminotransferases^10^ such as asparagine synthetase (*AsnS*) which leads to the formation of asparagine (Asn) and Glu from glutamine (Gln) and aspartate. Another enzyme, the NADH–glutamate dehydrogenase (*GDH*), plays its role in mitochondria, and is able to incorporate ammonium into Glu, in response to high levels of ammonium under stress^1^. The products of the primary metabolism, can then serve for the biosynthesis of other N-containing compounds.

High-throughput sequencing technologies have gained trust and reliability in the last decade representing the most advantageous and economical tool for deeply exploring genomes, transcriptomes, proteomes and metabolomes^11^. In particular, the transcriptomic approach has been widely used recently thanks to its high sensitivity and reproducibility in a number of different species (model and non-model) and experimental conditions^12–14^. Studies concerning cereal responses to nitrogen starvation have been also carried out partially unveiling differential expressed genes (DEGs) involved in these processes for instance in sorghum, rice, barley, and wheat^15–18^. However, the mechanisms regulating N use efficiency (NUE) in wheat (bread and durum) need to be further investigated. From several studies it is known that at molecular level different biological processes are involved in N stress and the expression of many crucial genes is altered in this particular condition^18–22^. Durum wheat [*Triticum turgidum* subsp. *durum* (Desf.) Husn.] crop is fundamental for the production of pasta and semolina^23^. It is the most widespread crop in the Mediterranean countries, holding an important role for their economies and traditions. These areas, including southern Europe and northern Africa, are characterized by low-rainfall, with critical problems like drought, salinity and low inorganic matter, causing limitations on growing crops without nitrogen fertilizers or biological nitrogen fixation through legumes^24, 25^. Farmers tend to increase sustainable agriculture, practicing rotation and limiting fertilization due to the high costs related to an overall poor and highly fertilizer-demanding environment. Being nitrogen directly linked with yield and quality (protein content), it is thus important to investigate how plants cope with N deficiency during plant growth and, specifically, during the process of grain development and senescence^26^. In this developmental stage, nitrogen plays a particularly important role, in fact low levels of nitrogen supply induce early senescence that is associated with a reduction of protein accumulation in wheat grain^27^.

From an -omics point of view, the grain filling stage still needs to be deeply investigated and genetic, metabolic and physiological data should be integrated for understanding how plants respond to reduced fertilizer inputs, possibly suggesting new targets for plant breeding.

In this study, using an RNA-sequencing approach, we investigated transcriptomic responses to chronic N-starvation in plants of durum wheat cv. Svevo, at the grain filling stage. To our knowledge, this is the first transcriptome-wide study on response to chronic N starvation in durum wheat. Our results represent a solid resource to clarify and further investigate the mechanisms regulating nitrogen use efficiency in this important crop, and to perform comparative transcriptomics in durum wheat germplasm showing different responses to N stress.

## Materials and Methods

### Plant growing and phenotypic analysis

Seeds of durum wheat cultivar Svevo were surface sterilized with sodium hypochlorite (0.5%, vol/vol) for 20 min, rinsed thoroughly in sterile water, vernalized at 4 °C for two weeks and then transferred to a hydroponic growth system. Seedlings were grown under 14 h/day illumination at 20 °C in net pots containing three plants each. Hydroponic solution was prepared starting from tap water (containing 13, 2, 26, 14, 2.6, 14 and 63 mgl^−1^ of Cl^−^, NO_3_-N, SO_4_-S, Na^+^, K^+^, Mg^2+^ and Ca^2+^, respectively) and using the salts reported in Yin *et al*.^28^. Two sets of plants were grown in two different conditions: standard nitrogen nutrition (control) including 2 mM Ca(NO_3_)_2_*4H_2_O, and N stress condition (stressed), with 0 mM Ca(NO_3_)_2_*4H_2_O. The whole root system was maintained submersed in the nutrient solution, which was continuously aerated with air-pumps and replaced every two days. The solution was maintained at pH 6.0 with 0.1N H_2_SO_4_.

The following phenotypic traits were collected on plants in the control and stressed conditions at the late milk developmental stage (Z77)^29^: plant height (PH), flag leaf area (FLA); number of culms per plant (NCPP); number of spikelets per spike (NSPS); number of kernels per spike (KNPS); kernel weight per spike (KWPS); flag leaf (FLDM) and spike (SDM) dry matter. Total nitrogen content in percentage was determined in roots, leaf/stem, flag leaf and spike tissues using a CHNS Analyser.

At the same Z77 stage, root, leaf/stem, flag leaf and spike tissues were sampled from three plants for each condition, and stored at −80 °C for RNA extraction.

### Illumina sequencing and quality control

Total RNA was extracted from each sample using the RNeasy Plant Mini Kit (Qiagen, Valencia, USA) and treated with DNaseI (Qiagen) according to the manufacturer’s specification. RNA concentrations were determined using NanoDrop® ND-1000 spectrophotometer (Thermo Scientific, Wilmington, USA) and its integrity was verified on a 1.5% agarose gel in 1X TAE/DEPC water. cDNA was synthesized from one microgram of total RNA using the QuantiTect Reverse Transcription kit (Qiagen), and sent to IGA (Udine, Italy) for processing. Twenty-four cDNA libraries (three biological replicates from each tissue in control and stress conditions) were subsequently prepared for an RNA-seq experiment with the Illumina High-Seq 2000 platform providing 100-bp paired-end reads (IGA). Trimming and clipping were performed with Trimmomatic-0.33^30^ using the following parameters: HEADCROP:10, LEADING:35 and TRAILING:35 MINLEN:25. The quality assessment was based on the remaining reads using the FASTQC quality control tool version 0.10.0 (http://www.bioinformatics.babraham.ac.uk/projects/fastqc).

### Read mapping and differential expression analysis

In order to map each cleaned library to the wheat genome, the bread wheat complete sequence, consisting of 23 pseudomolecules, along with the corresponding annotation file, were downloaded from EnsemblGenomes (ftp://ftp.ensemblgenomes.org/pub/plants/release-26/fasta/triticum_aestivum/dna/). STAR program^31^ was used for read mapping with outFilterScoreMinOverLread parameter set as 0.3. Gene expression levels were calculated with Cufflinks^32^ using geometric normalization and per-condition dispersion method by quantifying the Illumina reads according to the FPKM (fragments per kilobase per million mapped fragments). These values were used to perform a principal component analysis (PCA) and to check experimental and control biological replicates. Fold-changes were reported as the log (base 2) of normalized read count abundance for the N-stressed samples divided by the read count abundance of the control samples. Each dataset obtained from each considered tissue was filtered according to fold change values ≥1.5 and ≤−1.5.

### Transcript classification and Gene Ontology (GO) term analysis

Filtered data were used to identify and classify transcription factors and protein kinases by family, with iTAK (Plant Transcription factor & Protein Kinase Identifier and Classifier) software (http://bioinfo.bti.cornell.edu/cgi-bin/itak/index.cgi). Gene ontology terms were examined with agriGO^33^ for GO enrichment with custom annotation. For this analysis, the following parameters were chosen: hypergeometric statistical test method, multi-test adjustment hockberg FDR, significance level: <0.05 and 3 minimum number of mapping entries. Significant values were sorted by enrichment score (Query_item/Query_total)/(Background_item/Background_total) and GO redundancy was removed with REVIGO tool^34^. Heatmaps and hierarchical clustering were generated with MeV software^35^.

### Quantitative real-time PCR

To validate the reliability of the expression profiles observed in the RNA-seq data, 10 genes were selected for quantitative real-time PCR (qPCR) analyses using iTaq SYBR Green supermix (Bio-Rad, Munich, Germany). The RNase L inhibitor-like protein gene, RLI^36^ was used as an internal control. RNA material from the same samples used for RNA-seq experiment was used for this validation. Gene specific primers, designed by Primer3, are listed in Supplementary Table S1. The specificity of each primer was monitored by high-resolution melt analysis and by Sanger sequencing. The relative expression value was calculated by the delta-delta CT method and expressed as the fold change referred to the expression in the control (N 2 mM) plants (expression = 1)^37^. Three biological and three technical replicates per sample were analysed to ensure statistical reliability.

## Results

### Phenotype observations

Due to its high protein content^38^, durum wheat cultivar Svevo was selected for an RNA-seq experiment aimed at exploring the molecular response to chronic nitrogen starvation. Plants were grown under N standard or deficiency supply up to Z77 developmental stage. Nitrogen shortage caused serious changes in the phenotype, accelerating plant flowering time and senescence. As awaited, the whole plant growth was severely affected under nitrogen limitation^39^: all the traits considered sensibly differed between plants grown in the two conditions (Table 1). In particular, stressed plants were shorter than control ones and the flag leaf area was sensibly reduced (68%), affecting also grain filling and yield^27, 40^. This is consistent with a comparable significant reduction in the flag leaf dry matter. The most striking difference between the two groups of plants was, as expected, tiller number and development. In fact, while in the controls the average number of culms per plant was ≅4, the N starved plants generally did not tiller and showed a single culm. Consequently, also the yield traits were negatively influenced by N starvation. A significant reduction between normal and stressed plants was observed for the number of spikelets per spike (NSPS, over 34%), spike dry matter (SDM over 62%), and number of kernels per spike (KNPS, 75%). The average kernel weight per spike (KWPS) was 0.65 g in the control and 0.16 g in the stressed plants, with a reduction of ≅76% (Table 1). These results strongly support the previously formulated hypothesis that, when nitrogen is limited, a premature leaf senescence occurs affecting the rate and duration of protein accumulation in the seeds^41^.

**Table 1:** Phenotypic analyses on plant groups (control and stressed) of durum wheat grown with different N concentrations.

	Control	Stressed	% RR
PH (cm)	54.25 ± 2.67	44.09 ± 1.78	18.73
FLA (cm^2^)	16.73 ± 1.65	5.28 ± 0.81	68.44
FLDM (g)	0.061 ± 0.002	0.019 ± 0.001	68.85
NCPP	4.14 ± 0.4	1 ± 0.01	75.86
NSPS	24.6 ± 1.21	16.09 ± 1.09	34.65
SDM (g)	0.37 ± 0.002	0.14 ± 0.04	62.16
KNPS	20 ± 1.155	5 ± 0.58	75
KWPS	0.65 ± 0.034	0.15 ± 0.02	76.49

For each parameter, mean values (±standard error) and relative reductions between control and stressed conditions are presented.

PH: plant height; FLA: flag leaf area; NCPP: number of culms per plant; NSPS: number of spikelets per spike; SDM: spike dry matter; FLDM: flag leaf dry matter; KNPS: kernel number per spike; KWPS: kernel weight per spike; RR: relative reduction.

**Figure 1:**
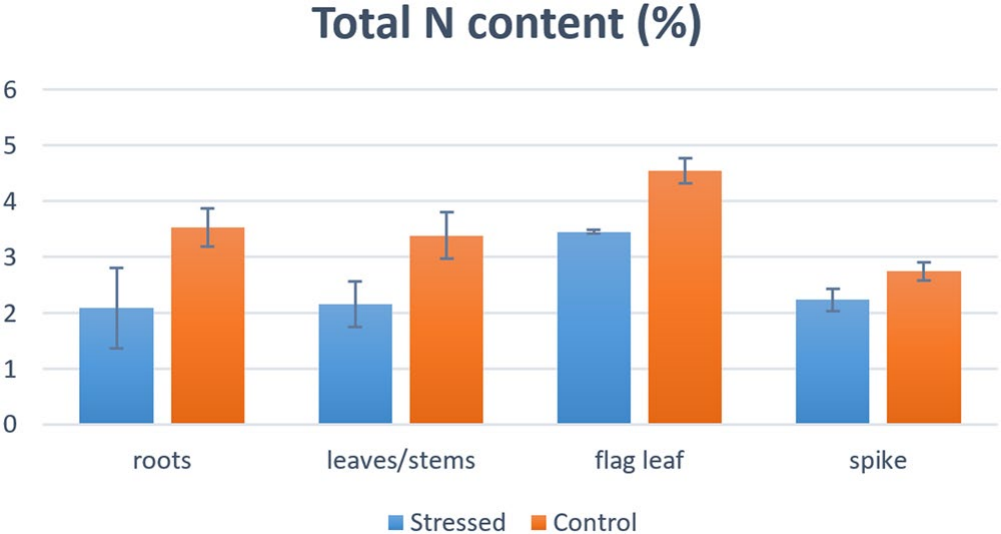
Total nitrogen (N) content detected in four tissues from durum wheat plants grown under standard (control) or N starvation (stressed) conditions. Values represent the N percentage relative to dry matter.

In addition, total nitrogen content was determined in the four collected tissues of the two plant groups. The relative N reduction was higher in the roots of N starved plants (≅41%) and decreased towards the top of the plant, with ≅24% reduction in the flag leaf and ≅18% in the spike (Fig. 1), as already observed in bread wheat^15^.

### RNA-seq statistics and differentially expressed genes

At the Z77 stage, root, leaf/stem, flag leaf, and spike tissues from control and from N stressed plants were used to prepare 24 cDNA libraries (4 tissues*2 conditions*3 biological replicates). High-throughput sequencing and subsequent read trimming/clipping delivered 25.20 million (M) reads for root, 30.08 M for leaf/stem, 27.85 M for flag leaf, and 26.63 M for spike tissues. The generated reads were then mapped against the bread wheat draft genome (EnsemblGenomes release-26). Varying contents of uniquely mapped reads versus multiple mapped reads were observed in the different tissues. The average of uniquely mapped reads percentage was higher in root (≅53%) with a corresponding low percentage of multiple mapped reads (≅12%). A lower value of uniquely mapped reads was observed in spikes (≅40%), dropping to ≅24% in flag leaf and leaf/stem tissues. Concomitantly, a higher value of multiple mapped reads was observed in spikes (≅30%), flag leaf and leaf/stem tissues (≅48% and ≅51%, respectively). Transcript profiles of the RNA-seq data were analyzed by calculating the read fragments per kilobase per million mapped reads (FPKM). PCA output highlighted the presence of outlier transcripts misleading the analysis for each tissue. These loci were thus removed from the annotation file before performing the mapping analysis. In summary, the number of loci considered for the differential expression analysis was 64991 for root, 64989 for leaf/stem, 64990 for flag leaf and 64989 for spike samples.

**Figure 2:**
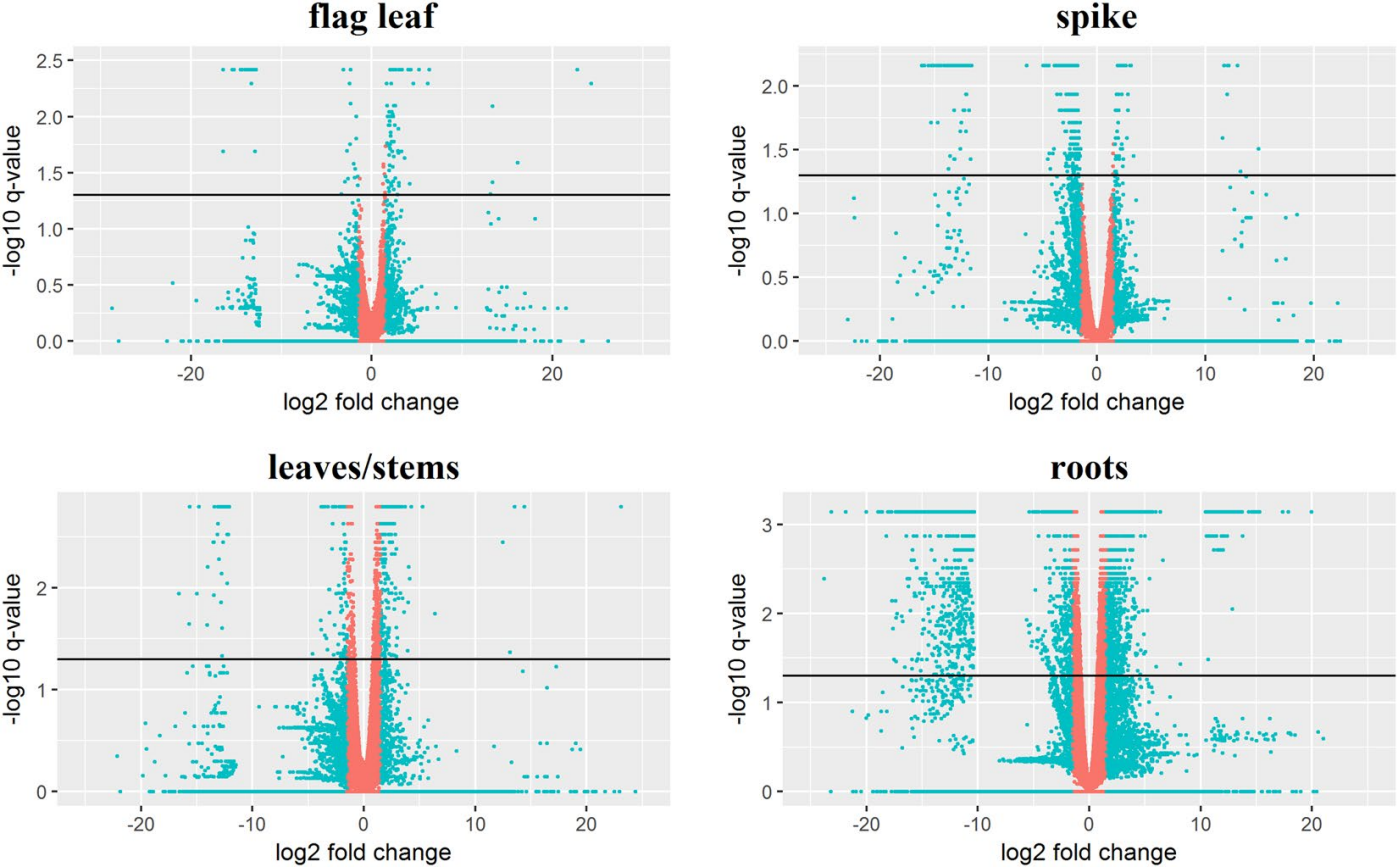
Volcano plot of the differentially expressed genes (DEGs) for each tissue investigated in this work. The y-axis corresponds to the mean expression value of -log10 (q-value), and the x- axis displays the log2 fold change value. The light blue dots represent up and downregulated DEGs, the pink dots denote not DEGs. The black bar indicates the q-value filtering.

**Figure 3:**
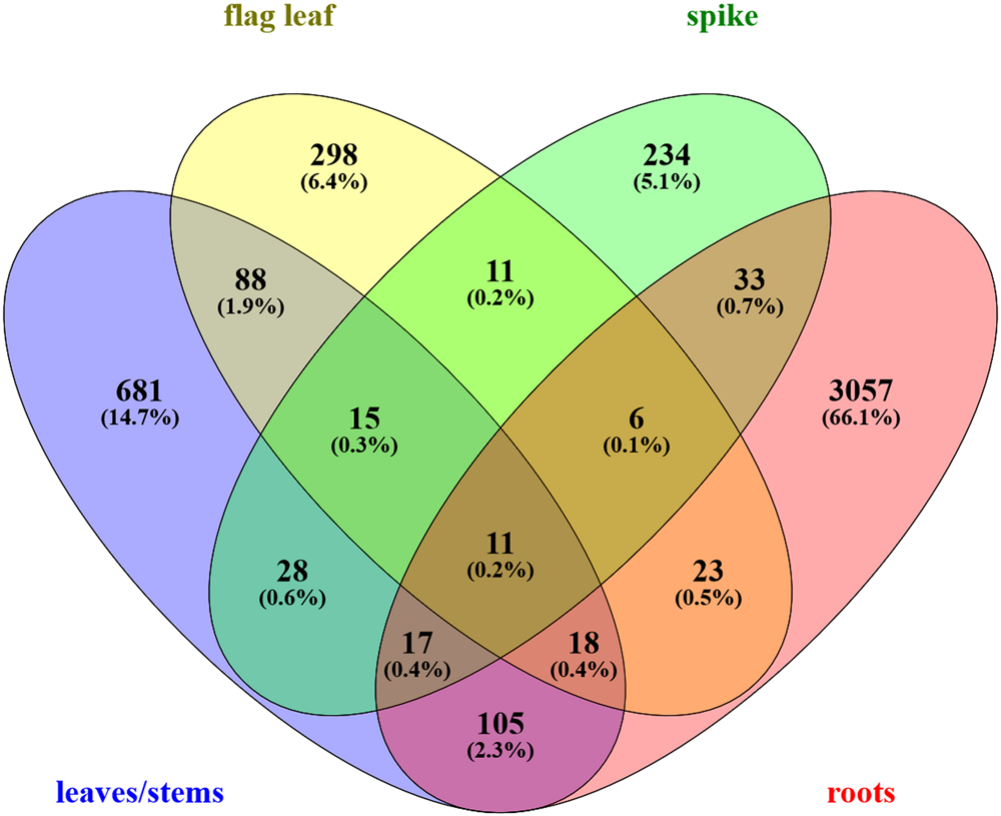
Venn diagram showing overlap of differentially expressed genes between or among tissues. Root, leaves/stems, flag leaf and spike RNA libraries were obtained from durum wheat, Svevo cultivar, grown in nitrogen starvation (0 mM nitrogen), or in standard (2 mM nitrogen) conditions.

Among the total of differentially expressed genes (DEGs) between control and N stressed samples, 3270, 973, 470, and 355 were identified in root, leaf/stem, flag leaf and spike tissues, respectively (p ≤ 0.05 and fold change ≥|1.5|, Table 2 and Fig. 2). All DEGs were annotated according to bread wheat available annotations, and to *Arabidopsis thaliana* for those lacking annotation (Supplementary Table S2). A Venn diagram was constructed to highlight uniquely and common genes among tissues (Fig. 3). Most of the uniquely DEGs were present in roots (66.10%), followed by leaves/stems (14.70%), flag leaf (6.40%), and spike (5.10%). Most shared genes were between root and leaves/stems (105), followed by leaves/stems and flag leaf (88).

**Table 2:** Differentially expressed genes (DEGs) and related enriched categories from GO analysis in response to nitrogen stress.

Tissue	up/down	DEGs	Filtered DEGs (log2FC)	GO enriched	Filtered GO (FDR)
*leaves/stems*	up	966	377	275	32
	down	904	586	566	186
*flag leaf*	up	206	144	156	9
	down	349	326	339	87
*spike*	up	64	60	71	25
	down	295	295	462	217
*roots*	up	2331	1411	754	114
	down	2340	1859	750	129

Number of total DEGs and number of DEGs filtered by log base 2 of the fold change (log2FC ≥ |1.5|) are shown. For each set of up- or downregulated genes, total GO categories identified from the enrichment analysis, and GO filtered by false discovery rate (FDR ≤ 0.05) are presented.

In order to validate the results obtained through RNAseq, we performed qPCR expression analyses in specific tissues on ten genes randomly chosen among N transporters, transcription factors, and main genes involved in N metabolism (Table 3), which showed a differential expression in the RNAseq experiment.

**Table 3:** RNA-seq results confirmed by quantitative qPCR.

Locus ID	Tissue	RNA-seq log2 (N−/N+)	RT-PCR log2 (N−/N+)	Putative function
	S	−12.87	−1.24	Probable peptide/nitrate transporter, ZIFL2
Traes_4AS_86FF10C36	R	2.13	1.13	Dof-type zinc finger DNA-binding family protein, DOF1.3
	L/S	2.38	3.96	
	FL	3.02	2.14	
Traes_5BL_CF5A8348D	R	1.59	NDE	Chaperone protein dnaJ 2, ATJ2
	L/S	2.54	4.87	
	FL	2.98	2.93	
	S	2.19	2.74	
Traes_4BS_E79CB87B7	R	−2.77	−3.79	ThiaminC, THIC
	L/S	−3.97	−1.84	
	FL	−2.77	−2.13	
	S	−6.51	−4.49	
Traes_1BL_241A3B9EF	R	1.81	1.85	Probable ubiquitin-conjugating enzyme E2 24,UBC24, Phosphate 2 (PHO2)
Traes_6DL_0A59B823C	R	3.21	1.09	Protein NRT1/PTR FAMILY 7.3, NPF7.3
Traes_2BL_309ED7C81	R	−1.56	−0.62	Glutamate dehydrogenase 2,GDH2
Traes_4BL_985CBED5D	R	2.23	1.14	Protein NRT1/PTR FAMILY 8.4,NPF8.4
Traes_2AS_A8CCC32D3	R	−1.59	−1.20	Protein TIFY 10B,TIFY10B
Traes_6AS_AB7CDF374	R	1.92	0.76	Nitrate reductase [NADH] 1,NIA1

R: root, L/S: leaves/stems, FL: flag leaf, S: spike, NDE: Not Differentially Expressed.

Results from qPCR analyses evidenced that expression trends of these genes were comparable to the ones obtained by means of RNAseq analysis (correlation coefficient R = 0.7086), thus validating the sequencing experiment (Table 3).

### GO classification and enrichment analysis of DEGs related to N metabolism

Out of a total of 3373 GO terms identified, 1256 and 2117 were associated to the significantly upregulated and downregulated genes in all tissues, respectively. When the FDR filter was applied to the list of differentially expressed genes, 799 GO terms were identified of which 180 among the upregulated and 619 among the downregulated genes (Table 2).

**Figure 4:**
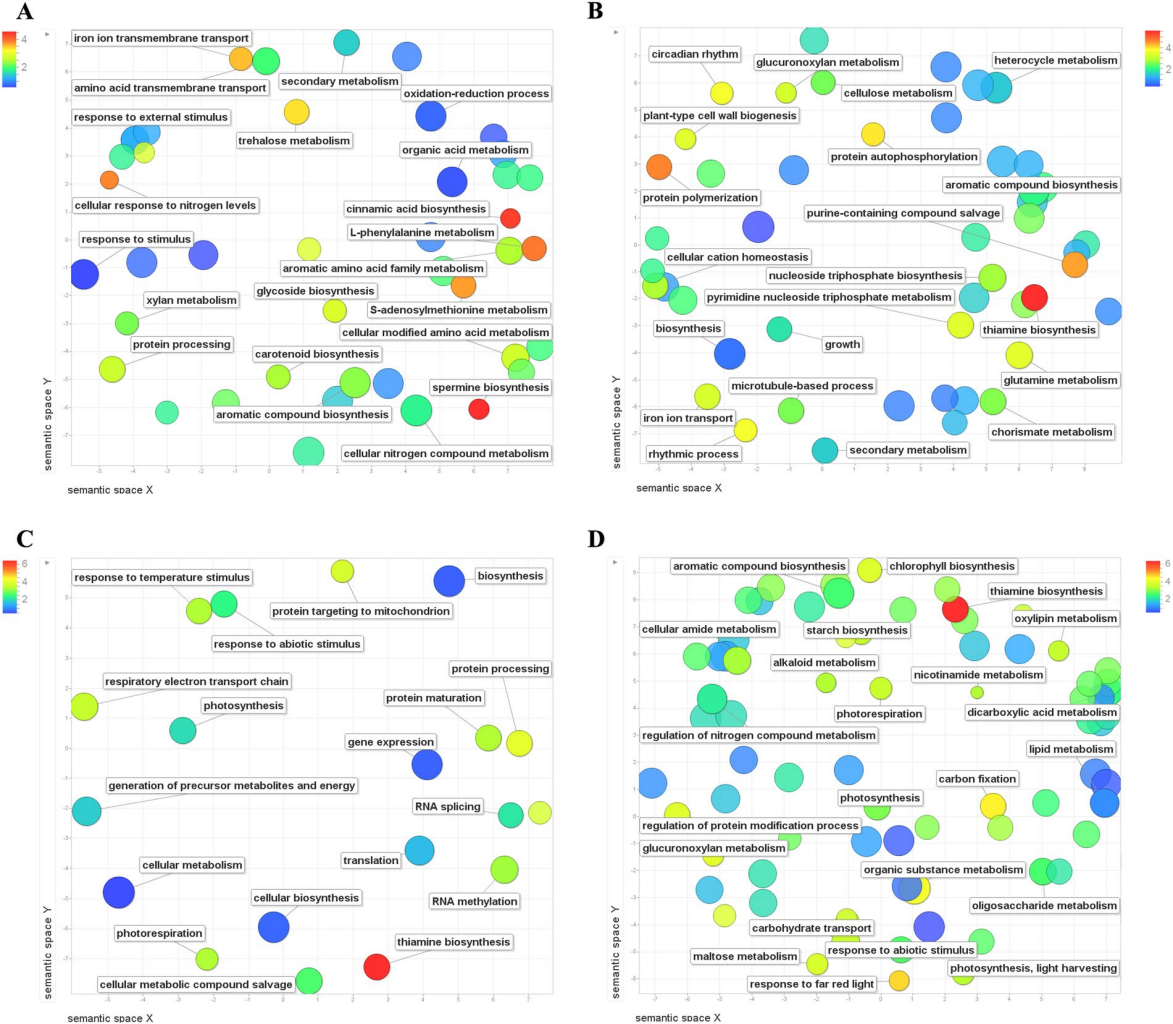
REVIGO semantic diagram summarizing enriched GO terms for roots (**A**), leaves/stems (**B**), flag leaf (**C**), and spike (**D**). Similar GO terms are placed close together in the plot. Bubble color indicates the log2 of the enrichment value for each category; circle size indicates the frequency of the GO term in GO database.

For summarizing GO categories belonging to each specific tissue, a semantic similarity scatter plot was obtained (Fig. 4). Starting from roots (Fig. 4A), we observed high values for categories involved in cellular response to N levels and cellular N compound metabolism, spermine biosynthesis, cinnamic acid and L-phenylalanine metabolism. Amino acid transmembrane transport and iron transport were also visualized in this graph, as well as trehalose and carotenoid metabolisms.

In leaf/stem tissues (Fig. 4B), thiamine biosynthesis covered the highest values, along with purine-containing compound salvage and protein polymerization. Several categories addressing nucleoside metabolisms were involved in these tissues, including glutamine metabolism along with others already described, such as cell wall biogenesis and cellulose metabolism. In flag leaf (Fig. 4C) we observed high enrichment values addressing photorespiration, respiratory elector transport chain, photosynthesis, response to abiotic stimulus and thiamine biosynthesis. Much more complex and articulate was the situation for spikes (Fig. 4D) in which, along with remarkable values addressing thiamine biosynthesis followed by several categories for carbon metabolism and photosynthesis, we also observed categories addressing regulation of N compound and cellular amide metabolism, aromatic compound biosynthesis, and alkaloid metabolism. Starch biosynthesis category was also remarkably enriched.

### DEGs directly involved in nitrogen metabolism

**Figure 5:**
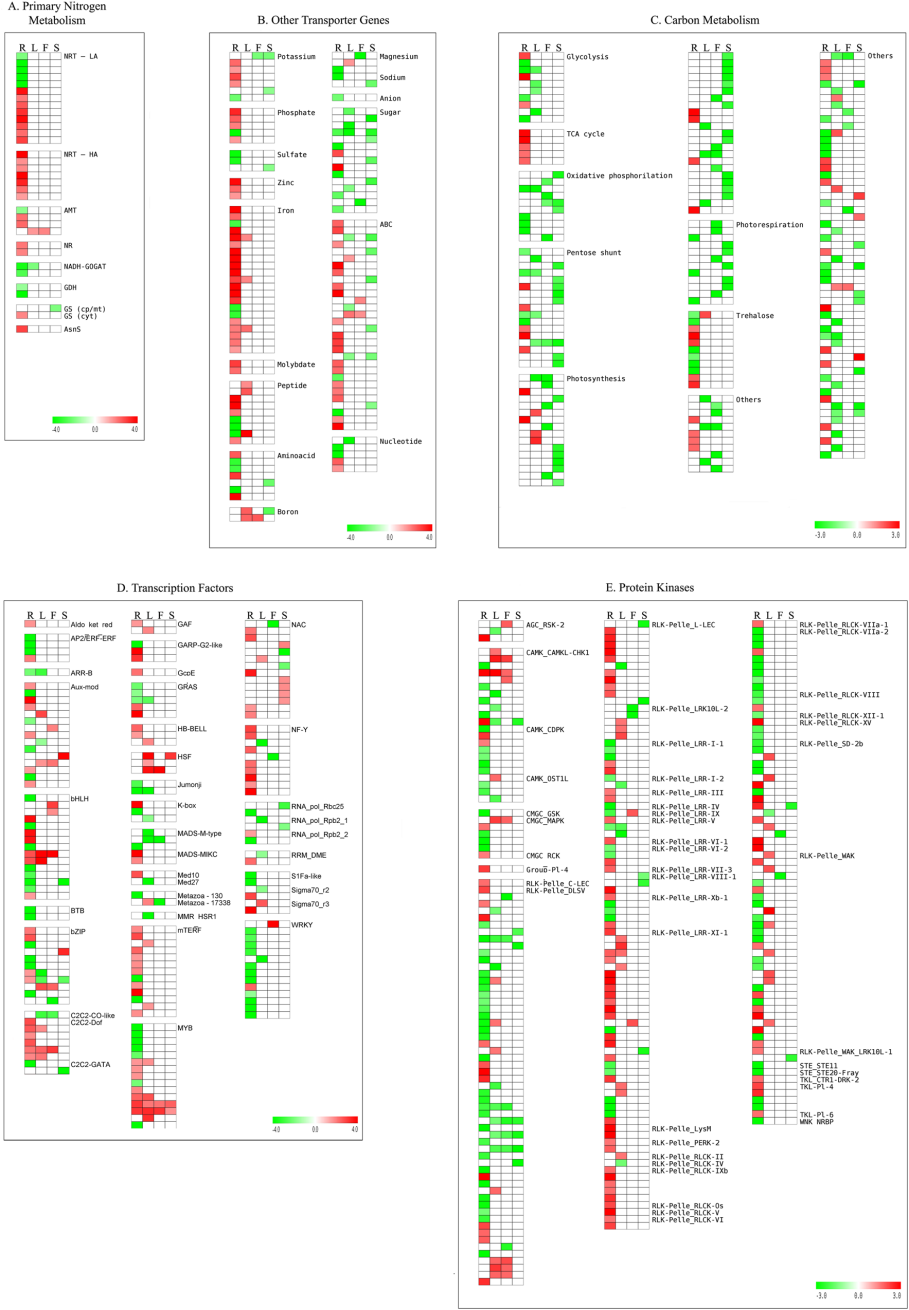
Heatmaps showing the expression patterns of genes involved in nitrogen stress response in durum wheat. Genes are grouped in five categories (**A** to **E**). Colours indicate the differential gene expression in nitrogen stressed tissues compared to non-treated plant tissues; green: downregulated, red: upregulated, white: not differentially expressed. R: roots, L: leaves/stems, F: flag leaf, S: spike.

Many genes involved in N absorption and assimilation were differentially expressed under N-stress compared to normal condition. Here, 12 DEGs encoding nitrate transporters were detected (Fig. 5A Supplementary Table S3). Two genes were downregulated while the other ten showed increased expression under N-stress. Two of these transporters are orthologous to the ones differentially expressed in N stress conditions in barley, recently described^18^. Among the DE transporters responsive to ammonium, two orthologues to AMT3-1 from barley were upregulated in durum wheat stressed roots, while one orthologue to Arabidopsis AMT2 was downregulated in stressed roots and one orthologue to Arabidopsis AMT1-4 was upregulated in both stressed flag leaf and leaf/stem tissues (Fig. 5A, Supplementary Table S3).

The key genes involved in nitrate assimilation were found differentially expressed mostly in root tissues under N stress conditions. *Nitrate reductase* [NADH] *1*, along with *Asparagine synthetase*, and the cytosolic *Glutamine synthetase* genes were found upregulated in roots (Fig. 5A, Supplementary Table S3). On the contrary, the chloroplast/mitochondrial *Glutamine synthetase* gene appeared downregulated in spikes. Durum wheat genes orthologous to *Arabidopsis* chloroplast *Glutamate synthase 1* [NADH-GOGAT 1] were downregulated in response to N-stress in root and leaf/stem tissues. A downregulation was observed for genes orthologous to three members of the *Glutamate dehydrogenase* family from *Arabidopsis*, in response to N-stress (Fig. 5A, Supplementary Table S3).

### Other DE transporter genes

The expression of many genes associated with absorption or translocation of other nutrients also changed under N stress. Seven potassium transporters were differentially expressed, including five members of the POT family, which were mostly upregulated in roots and downregulated in flag leaf and spike (Fig. 5B, Supplementary Table S3). Out of five differentially expressed phosphate transporters, two orthologues to *PhO1* and one to *RHS15* were upregulated and one strongly repressed in stressed roots. A considerable number of DEGs was found for ABC transporters (30), which were mostly upregulated in roots. Iron transporters (21) included orthologues to *NRAMP1*, *VIT1*, *YSL2,6,8* from *Arabidopsis* and were generally upregulated in roots. Transporter genes for sugar (15) were mostly downregulated in roots, but also in other tissues. Other DEGs included transporters for peptide (9), aminoacid (7) sulfate (3), zinc (3), and molybdenum (2, Fig. 5B, Supplementary Table S3).

### Carbon metabolism

A general alteration was observed for genes participating to carbon metabolism, especially for those involved in glycolysis, tricarboxylic acid (TCA) cycle, oxidative phosphorylation, pentose phosphate pathway, photosynthesis and photorespiration. Most of the genes involved in glycolysis were downregulated under N starvation, including orthologues to phosphoglycerate kinase (*PGK*) and to biphosphoglycerate mutase (*BPGM*), which were found strongly downregulated in leaves/stems and roots, respectively (Fig. 5C, Supplementary Table S3). However, among the few upregulated genes, an orthologue to enolase (*ENO*) was found in roots. Several genes belonging to TCA cycle were upregulated in roots, such as wheat orthologues to malate dehydrogenase (*MDH*) and succinate dehydrogenase (*SDH*). A diffused downregulation was observed for genes involved in oxidative phosphorylation, for instance, the orthologues to NADH oxidoreductases (e.g. *ndhF*, *ndhD*) and ATP synthases (e.g. *atpC* and *atpI*) (Fig. 5C, Supplementary Table S3). Pentose phosphate pathway DEGs identified in spike tissues were downregulated, while a slight majority of those identified in roots were upregulated under N stress. Among these, the genes orthologous to glucose 6-phosphate dehydrogenase (*G6PD2*) and 6-phosphogluconate dehydrogenase (*6PGD*) were both downregulated in roots and leaves/stems, whereas the orthologues to ribose-5-phosphate isomerase (*RPI*) were upregulated in roots. The majority of DEGs involved in photosynthesis was found in spike, while a smaller number was identified in the other tissues. DEGs identified in spike and flag leaf were all downregulated, especially the genes related to photosystem I and II (i.e. *psaO*, *psaK*, *psbZ*, *psbE*, *psbK*) (Fig. 5C, Supplementary Table S3). Few genes identified in roots were upregulated, including the 4-hydroxy-3-methylbut-2-enyl-diphosphate synthase (ferredoxin) gene (*ISPG*). DEGs for photorespiration were all downregulated, for example, the orthologues to NADH dehydrogenase in flag leaf, while the ribulose bisphosphate carboxylase small chain (*RBCS*) gene in root, flag leaf and spike tissues. Among the other genes involved in carbon and energy metabolism, the orthologues to several genes of the sucrose metabolism were upregulated in roots, including sucrose-phosphate synthase 1 (*SPS1*), sucrose synthase 3 (*SUS3*), trehalose-6-phosphate synthase (*TPS1*), and three starch synthase genes (*SS2*, *SS3*, *SS4*).

### DE transcription factors and protein kinases

Due to N starvation, 170 unique genes encoding transcription factors were differentially expressed. Among them, 125 were DE in root, 40 in leaves/stems, 16 in flag leaf, and 18 in spike. The TF genes identified belonged to different families, including MYB (15), bHLH (15), bZIP (11), WRKY (14), mTERF (13), NAC (14), C2C2-Dof (6), NF-Y (10), the auxin-modulated ARF (8) and AUX/IAA (8), and other families (Fig. 5D, Supplementary Table S3). Several TF families were preferentially expressed in specific tissue(s). For instance, most MYB genes were differentially expressed in roots, and NF-Y DE genes were all upregulated in N stressed roots. Additionally, most members (11) of the WRKY family were downregulated in roots, whereas C2C2-Dof TFs were always upregulated in durum wheat root, leaf and stem, and flag leaf tissues following N stress (Fig. 5D, Supplementary Table S3).

In this study, 250 unique protein kinases (PK) were identified in all tissues. In root, 178 genes for protein kinases were found, 88 of which were upregulated and 90 downregulated. In leaves/stems, out of 41 PK genes, 27 were upregulated and 14 were downregulated, while in flag leaf tissues, out of 21 PK genes, 10 were upregulated and 11 were downregulated. In spike tissues, 10 genes for protein kinase were detected, all of which were downregulated. As expected, the largest number of PKs belonged to receptor-like kinase (RLK)/Pelle family, massively expanded in flowering plants and representing more than 60% of the PKs^42^. We found 208 PKs belonging to this family with a remarkable number (55) of PKs carrying leucine-rich repeat (LRR) domains (Fig. 5E, Supplementary Table S3). Three PKs belonged to AGC (PKA–PKG–PKC), 22 to CAMKs (calcium- and calmodulin-regulated kinases), seven to CMGC (including cyclin-dependent kinases, mitogen-activated protein kinases, glycogen synthase kinase and cyclin-dependent kinases), two to STE (including many kinases functioning in MAP kinase cascades), six to TKL (tyrosine kinase-like kinases), and one plant-specific kinase (belonging to Group-Pl-4) and one WNK_NRBP.

### Other DE genes

Nitrogen deficiency causes several plant stress responses. In this study, a number of genes related to detoxification and protection from oxidative stress were upregulated, the majority of which were found in roots. A total of 17 orthologues to Glutathione S-transferases (*GSTs*) were counted in the DEG dataset, 12 of them were identified in roots, while for leaves/stems and flag leaf only three and two were found, respectively. Particularly, eight *GSTs* were upregulated, two in leaves/stems (*GSTL2*) and six in roots (*GSTZ1*, *GSTU24*, *GSTF13*). In addition, 16 orthologues to cytochrome P450 (*CYP450*) were identified, eleven in roots (nine upregulated), four in leaves/stems (two upregulated) and one in flag leaf (upregulated). Five orthologues to genes involved in the carotenoid/abscisic acid biosynthesis pathway were found upregulated in roots: phytoene synthase (*PSY1*), phytoene desaturase 3 (*PDS*), carotenoid isomerase (*CRTISO*), beta-carotene 3-hydroxylase 2 (*BETA-OHASE 2*) and zeaxanthin epoxidase (*ZEP*). Orthologues genes protecting plant cells from oxidative damage were also identified. Catalase 2 (*CAT2*), superoxide dismutase [Fe] 2 (*FSD2*), aldehyde dehydrogenase (*ALDH*) and cinnamyl-alcohol dehydrogenase 1 and 4 (*CAD1*, *CAD4*) were upregulated in roots, while methionine sulfoxide reductase B1 (*MSRB1*) was upregulated in leaves/stems.

The orthologues of homocysteine S-methyltransferase (*HMT*) and Met S-methyltransferase (*MMT*), involved in the S-methylmethionine (SMM) cycle, were also upregulated. The former was upregulated in all tissues analyzed (*HMT1* in roots and *HMT2* in the other tissues), while the latter was upregulated only in roots. Among other DEGs, we also evidenced hormone signaling genes, such as auxin response factor 2 and 18, and the important coenzyme pyridoxal phosphate (PLP)-dependent transferase, all upregulated in roots (Supplementary Table S3).

## Discussion

The aim of our study was to investigate the transcriptomic response of durum wheat to N starvation during the grain filling. Instead of analyzing the immediate response to this condition, we focused on genes found differentially expressed after a long-term N stress, and thus representing the cumulative response to N nutrition and availability during the plant cycle until the late milk stage. We used the variety Svevo, which is reported to have a good N use efficiency, and analyzed DEGs in all major organs of the plant, namely roots, leaves/stems, flag leaf and spike in stressed plants compared to plants grown in normal N supply conditions. To our best knowledge, this is the first study on transcriptomic response of durum wheat under N stress, and especially under chronic starvation conditions.

Plants were affected by N shortage primarily on size, showing almost no tillering, decreased height and lower flag leaf area and dry matter. This overall plant depression was also evident in the spikes and in the number of seeds per spike, while the average seed weight was comparable, thus indicating that the stressed plants concentrated all their energy to produce viable seeds.

As observed for the relative reduction of total N content, also the number of DEGs decreased from the roots to the top of the plant. In fact, most DEGs were found in root, which was the main responsive part of the plant to N stress. Gene ontology analysis helped highlight the biological processes mostly involved in the response to this condition. Cellular response to N levels was a highly addressed GO category in roots, comprising several upregulated genes involved in N absorption and assimilation. In above ground tissues, other processes including photosynthesis and photorespiration were highly influenced by N stress. The relation between carbon and nitrogen metabolism has been in fact widely shown, implying alterations in plant growth, physiology and development^7, 20^.

Two types of transporters, low- and high-affinity nitrate transporters (NRT), carry out nitrate absorption in plant roots. In this study, the N starvation condition led to a marked reduction in total N content in roots, with a parallel upregulation of several genes encoding both low- and high- affinity NRTs. A higher activity of NRTs may be part of the durum wheat adaptation to enhanced N uptake due to N shortage. Among high- affinity NRTs, the gene orthologous to Arabidopsis *NRT 2.5* was strongly upregulated in durum wheat. In sorghum, N stress tolerant genotypes showed higher abundance of the transcripts *NRT2.2*, *NRT2.3*, *NRT2.5*, and *NRT2.6*, related to high affinity NRTs^16^.

Nitrogen accumulation in wheat occurs in various organs, but predominantly in leaves, since these are more efficient in N remobilization. In particular, N remobilization from flag leaf was positively correlated with N yield per spike and per area^43^. In N starvation conditions, we observed the upregulation of two genes encoding for oligopeptide transporters in the flag leaf and one in leaves/stems, suggesting that protein degradation might occur in these tissues, with subsequent transportation of N accumulating compounds, such as peptides. Under low N supply, higher N remobilization has been observed compared to high N supply^44^. This could explain the lower drop in total nitrogen content in the organs mainly involved in N remobilization, that is flag leaf and spike. Considering this basis, our results are in agreement with a metabolic and transcriptional analysis in barley, where it was found that members of amino acid/peptide transporter families are essential components in N remobilization^45^. Changes in the expression of aminoacid/peptide transporter genes were also observed in roots, suggesting the activation of mechanisms to increase N uptake and accumulation aimed at contrasting N deficiency^16^. In cereal crops, not only leaves, but also stems, glumes and roots are considered as N sources for grain development, and N remobilization from these organs may also play an important role in grain filling as a buffering leaf senescence mechanism in the post-anthesis period^11^. This is in line with the observation that a number of proteases were upregulated in most of the tissues here analysed. Among them, the vacuolar processing enzyme gamma (*VPE-γ*), a possible regulator operating in the early stages of senescence, a putative metacaspase, an asparaginase, and one cysteine protease (papain family) were found upregulated in roots, while another cysteine protease (*SAG12*) was upregulated in leaves/stems. Interestingly, in common wheat low N plants at the anthesis stage, a cysteine protease of the papain family, involved in bulk degradation of stromal proteins during leaf senescence^46^, and *SAG12*, a marker of senescence and remobilization, were upregulated, the latter particularly in leaves and lower stems^15^.

Glutamine synthetase cytosolic isozyme 1 (*GS1*) has several metabolic functions, including primary ammonium assimilation in the roots and re-assimilation of catabolism-derived ammonia, for transport and distribution throughout the plant^47^. During leaf senescence, *GS1* is involved in the assimilation and recycling of the ammonia generated from catabolic processes^47, 48^, remobilizing N to developing grains in cereals^49, 50^. In this work, an upregulation of a *GS1* under N stress conditions was observed in the roots of Svevo. Although it is difficult to establish a global model of cytosolic GS response to variation in nitrogen availability in plant roots and leaves^47^, in *Arabidopsis* it has been observed that two cytosolic *GS* members were upregulated under severe chronic N stress^20^. The upregulation of *GS1* in durum wheat roots under N chronic stress might be related to the re-assimilation of ammonia released from protein degradation occurring under N deficiency^20^.

One member of the Asparagine synthetase (*AsnS*) gene family was upregulated in durum wheat roots under N stress. Asparagine contributes to N remobilization in plant tissues, and its synthesis occurs by transferring an amide group from glutamine to aspartate, thanks to asparagine synthetase^7^. Based on similarity analyses, we realized that the *AsnS* DEG here detected belongs to class III of AsnS family^51^. Recent evidence showed that the *AsnS* rice gene (*OsAS1*) belonging to class III (according to our phylogenetic analyses, data not shown) is mainly expressed in roots in an NH_4_^+^-dependent manner, similarly to *GS1* ^52^. The upregulation of *AsnS* in durum wheat following N deficiency might be due to a higher availability of ammonium, following protein degradation^20^.

Many iron transporter genes were activated in durum wheat roots under N stress. Iron plays a crucial role in N metabolism, being a metal cofactor of enzymes of the reductive assimilatory pathway, including nitrate reductase, nitrite reductase, and glutamate synthase^53, 54^. Moreover, the differential expression of transporter genes for other minerals and nutrients indicates that their uptake in durum wheat plants might be affected by N metabolism under cross-talking regulation.

We detected a high number of DE ABC transporters, mostly upregulated in durum wheat N stressed roots, two in flag leaf, and six downregulated in spike. ABC transporters have been shown to be involved in several processes such as phytate accumulation in seeds, transport of the phytohormones auxin and abscisic acid, peptides, sugars, lipids, etc., thus playing an important role in plant development and nutrition, organ growth, response to abiotic stress, and in plant interaction with the environment^55, 56^.

Nitrogen stress caused relevant alterations in the expression of genes related to carbon metabolism in durum wheat. It is well known that pathways for carbon and N assimilation are strongly related^57, 58^; in particular, for many species, N deficiency causes an increase in root growth due to a main redirection of carbon assimilates towards the belowground tissues^59^. Indeed, total carbon in our samples was higher in stressed tissues compared to control ones, especially in roots and stems (data not shown). An accumulation of C was also observed in low nitrogen common wheat stems, particularly found in the form of fructans^15^. Under N starvation, several enzymes involved in sucrose and starch metabolism were found upregulated in Svevo roots. This could be linked to a carbon partitioning oriented to root growth, since sucrose plays a role as a stimulant for lateral root formation^60^; recently it has been hypothesized a role for root starch in root development and in the response to nitrate availability^61^.

In durum wheat roots, the orthologues to malate dehydrogenase (*MDH*) and glyceraldehyde 3-phosphodehydrogenase (*GAPDH*) were upregulated. These genes are particularly relevant for the production of reducing equivalents, thus maintaining the glutathione reduced for the functioning of glutathione S-transferase (*GST*). *GST* catalyzes the glutathione-dependent detoxification reactions and the reduction of hydroperoxides. *GST*s may act as binding proteins that sequestrate flavonoids in the vacuole for protection against environmental stresses^62^. On the other hand, we observed a downregulation of the orthologues to genes involved in the oxidative pentose pathway, including those providing reducing power (NADPH). In particular, a downregulation was observed for the orthologues to glucose 6-phosphate dehydrogenase (*G6PDH*), which is the rate-limiting step in this process and the orthologue to 6-phosphogluconate dehydrogenase (*6PGD*), which was shown to be nitrate inducible in *Arabidopsis* ^63^.

Under prolonged N deficiency, leaf senescence is generally accelerated and ultimately plant growth can be inhibited^64^. One of the causes of these processes is the decrease in photosynthetic activity of the plant, which is primarily due to the breakdown of Rubisco^65^. In this work, the majority of DEGs involved in photosynthesis and photorespiration including those coding for proteins related to Photosystem I and II, ATP synthase and Rubisco were downregulated. Among the few upregulated genes, the orthologue to *ISPG*, was found upregulated in roots. It reduces Ferredoxin (Fd) in plastids and thus it is important for the functioning of *Fd-GOGAT* and therefore for ammonium assimilation^19^.

More than 30 TF families were identified to respond to N-starvation in durum wheat, among which MYB and bHLH genes were the most abundant. These two TF families interact to regulate target genes and several R2R3-type MYB and bHLH TFs have been reported to be involved in plant stress responses^66^. Some R2R3-type MYB proteins have a role in the regulation of phenylpropanoid pathway^67^, in fact over-expression of specific MYB genes (e.g. *PAP1*, found upregulated in the present study) enhanced accumulation of lignin and flavonoids^20^. In the present work, the expression of several Svevo MYB transcription factors was found to be influenced by N conditions; for instance, *MYB48* appeared to be upregulated in leaves/stems and root tissues of Svevo plants under N starvation. In rice, the overexpression of *OsMYB48-1* enhanced the tolerance toward abiotic stresses probably via the regulation of stress-mediated ABA biosynthesis^68^. Furthermore, *MYB86* and *MYB20*, which in the present study were downregulated in N stress conditions, were also associated with abiotic stress responses in *Arabidopsis* ^69, 70^. These MYB genes could also be regulated by miRNAs as it is the case of Svevo *MYB3*, which was identified as the target gene of ttu-miR319f^71^.

Another family of TFs DE in durum wheat under N starvation was the NF-Y TFs. Recent evidence in *T. aestivum* showed that NF-Y TFs are induced by low nitrogen conditions, increasing nitrogen uptake and grain yield^72^. In our study, NF-Y factors were mainly upregulated in the roots.

WRKY is one of the largest TF families responsive to N-deficiency in rice, with twelve WRKY members induced in the sheaths versus none in the roots^17^. In durum wheat, most of the WRKY identified to be responsive to N chronic stress were found in the roots. This difference in WRKY expression profiles between the two Graminaceae species might be due to the large number of WRKY transcription factors and their unknown and diverse roles under complex environmental stimulations^73^, and to the different plant developmental stage and environmental conditions used in the two species. Thanks to the relevant role they play in plant stress responses, WRKY proteins contribute to the establishment of complex signalling webs and are potential candidates for imparting N stress tolerance.

Moreover, MYB, NAC and WRKY TFs, in combination with hormones (ABA and jasmonic acid), have been shown to be involved in the transition of grain filling and developmental senescence^45, 74^.

Protein kinases are important for development and adaptation to abiotic stress in plants, by regulating transcription through the phosphorylation of transcription factors^75^. In the present work, different groups of PK genes were identified and many of them showed to be influenced by N deficiency. Differentially expressed PK genes were more abundant in root and leaves/stems, but were also identified in flag leaf and spike tissues. Most of the protein kinases belong to the RLK/Pelle family, which are involved in many different processes in plants, including signaling networks concerning abiotic environmental stimuli^76, 77^. Among the numerous RLK/Pelle DEGs, we found some Wall Associated Kinases (WAK) upregulated in roots, which might be involved in root growth under N limitation^78^.

Kinase genes related to the *CIPK23* were downregulated in Svevo roots under N starvation conditions. This observation is in agreement with studies in *Arabidopsis*, where it has been demonstrated that *CIPK23* is a nitrate-inducible protein kinase belonging to the family of CBL-interacting protein kinases being a negative regulator of the high-affinity nitrate transport response^79^. Therefore, the upregulation of high-affinity nitrate transporters already described here, may be a consequence of the downregulation of its repressors.

This study evidences an upregulation of several important genes for detoxification and protection from oxidative damage under N deficiency. A massive accumulation of reactive oxygen species (ROS) was also observed in *Arabidopsis* roots under N starvation^80^. In our study, several root DE orthologues to *GST* genes along with orthologues to *CYP450* and several other genes including catalase 2 (*CAT2*), superoxide dismutase [Fe] 2 (*FSD2*) and aldehyde dehydrogenase (*ALDH*) were observed. This finding might suggest a role of roots in maintaining the redox homeostasis^81^, in agreement with a number of studies in *Arabidopsis* ^20^ and rice^19, 56^.

As expected, an upregulation of several genes involved in abscisic acid biosynthesis was noticed. This phytohormone, along with auxin and cytokines, has been shown to be involved in N demand and acquisition by bringing changes in plant physiology and morphology^82^.

An activation of the S-methylmethionine (SMM) cycle has been suggested to be involved in stress adaptation, resulting in the final products of cysteine and several polyamines such as spermine, spermidine and putrescine^83^. Cysteine plays a critical role in protection against abiotic/biotic stresses thanks to its derivatives, such as GSH and phytochelatin polymers^84, 85^. Moreover, an upregulation of pyridoxal phosphate (PLP) coenzyme was observed; PLP is important for the functioning of several aminotransferases, thus playing a role in N metabolism, and is notably involved in several stress responses^86^.

Conclusively, our study provides a landscape of the genes differentially expressed in durum wheat root, leaves/stems, flag leaf and spike following chronic N starvation. The severe phenotypic changes observed in plants under N stress were reflected in an altered transcriptomic activity in all organs of the plant, but especially in roots. To our best knowledge, this is the first comprehensive transcriptomic analysis in durum wheat under N deficiency, and provides valuable resources for a better understanding of durum wheat response to this stress and subsequent improvement of N use efficiency.

## Electronic supplementary material

Supplementary Table 1

Supplementary Table 2

Supplementary Table 3
