# Supplementary material for: Transcriptomic response of durum wheat to nitrogen starvation
Source: Sci Rep. 2017 Apr 26;7:1176. doi: 10.1038/s41598-017-01377-0 (PMC5430780; doi:10.1038/s41598-017-01377-0)
Supplement: Supplementary file 1 — Supplementary Table 1 [file 41598_2017_1377_MOESM1_ESM.pdf]

Transcriptomic response of durum wheat to nitrogen starvation

Pasquale L. Curci[1*], Riccardo Aiese Cigliano[2], Diana L. Zuluaga[1], Michela Janni[3], Walter Sanseverino[2], Gabriella Sonnante[1*]

[1] Institute of Biosciences and Bioresources, National Research Council (CNR), Via Amendola 165/A, 70126 Bari, Italy

[2] Sequentia Biotech SL, Calle Comte D'Urgell 240, 08036 Barcelona, Spain

[3]Institute for Electronics and Magnetism, National Research Council (CNR), Parco Area delle Scienze 37/A, 43124 Parma, Italy

Corresponding authors

Gabriella Sonnante

Institute of Biosciences and Bioresources, National Research Council (CNR)

Via Amendola 165/A, 70126 Bari, Italy

Email: gabriella.sonnante@ibbr.cnr.it

Pasquale Luca Curci

Institute of Biosciences and Bioresources, National Research Council (CNR)

Via Amendola 165/A, 70126 Bari, Italy

Email: luca.curci@ibbr.cnr.it

Supplemetary Table S1. Primers used for RNA-seq validation experiment

Gene	Forward (5' - 3')	Reverse (5'-3')	Product length (bp)	Reference	EnsemblPlants
ZIFL2	CGTCTGCTCTACCTTGCCGA	CCTCCCATGCTTATCGGCCA	184		TRAES3BF102700140CFD_g
DOF1.3	GGTGATCCTCTCTGCACCCC	CTGCTGCAACTCTCTCCCTCG	223		Traes_4AS_86FF10C36
ATJ2	GAAGCTATCTCTTGCCCGCAA	GTAACCAGCACCTTGGCAGC	103		Traes_5BL_CF5A8348D
THIC	GACCGGAGATAGCGGGCATT	GGGCGACTTCWGAYCGGACA	215		Traes_4BS_E79CB87B7
Phosphate 2 (PHO2)	GAGGGCTTGCATTGTTGGGG	CCCTCCGGATTCCACACCTC	219		Traes_1BL_241A3B9EF
TaGS2_cino_F	GGACGATTTCGAAGCCAGTGGAG	ATGGCACTTGTGAAGTGACCTTG	261		Traes_2BL_342BDEA35
TaNPF7.1_F	CTACAAGACCTGCGCCATCTTC	GATGAGGTATAGCCGCGAGGAG	82	Buchner and Hawkesford (2014)	Traes_6DL_0A59B823C
TaGDH2	AGGATGGGAGCATTCACCTTGG	GGATATAAGAACTKTCATCCACCACG	138	Buchner and Hawkesford (2014)	Traes_2BL_309ED7C81
TaNRT2.1	GCCGCTTGTCTTCCACGCA	GTCCTTGGCCATGTCTCCCTTCT	181	Taulemesse *et al*. 2015	Traes_6DS_9184F1B4B.1
TaNRT1	CATGAAGGCTGGCTCTGGGGT	GAGCTGAGGAATCACATGGCAAAAC	190	Taulemesse *et al*. 2015	Traes_4BL_985CBED5D
Tae-TIFY10A	GTGTCTGCAGCAGTTGTGGTC	GGGAAGGTGCTCGTGTTCAAC	119	Zhang *et al*. 2014	Traes_2AS_A8CCC32D3
TaNR	GGCCAATTCYTTCATCTCCTTCTG	TACRTSCACAGATTGATGCGTCSA	120	Buchner and Hawkesford (2014)	Traes_6AS_AB7CDF374
RLI(a)	TTGAGCAACTCATGGACCAG	GCTTTCCAAGGCACAAACAT	84	Gimenez *et al*. 2011	Ta2776*

* = unigene
